# Evaluation of Ulcer Protective Activity of *Morus alba* L. Extract-Loaded Chitosan Microspheres in Ethanol-Induced Ulcer in Rat Model

**DOI:** 10.1155/2022/4907585

**Published:** 2022-09-30

**Authors:** Sarita Garg, Rajeev K. Singla, Md. Mominur Rahman, Rohit Sharma, Vineet Mittal

**Affiliations:** ^1^Department of Pharmaceutical Sciences, Maharshi Dayanand University, Rohtak, Haryana, India; ^2^Institutes for Systems Genetics, Frontiers Science Center for Disease-related Molecular Network, West China Hospital, Sichuan University, Chengdu 610041, Sichuan, China; ^3^School of Pharmaceutical Sciences, Lovely Professional University, Phagwara, Punjab 144411, India; ^4^Department of Pharmacy, Faculty of Allied Health Sciences, Daffodil International University, Dhaka 1207, Bangladesh; ^5^Department of Rasa Shastra and Bhaishajya Kalpana, Faculty of Ayurveda, Institute of Medical Sciences, Banaras Hindu University, Varanasi 221005, Uttar Pradesh, India

## Abstract

Due to an unhealthy lifestyle, gastric ulcers have become a very common disease these days. Moreover, the side effects linked with the prolonged use of conventional treatments have shifted the paradigm towards herbal therapies. The leaves of *Morus alba* L. (Family-Moraceae) have been traditionally used for a large number of metabolic diseases. In the present research, we focused on the development of chitosan microspheres using extracts of leaves of *Morus alba* L. and their evaluation for gastroprotective efficacy against ethanol-induced ulcers in experimental rats. The process of development of *M. alba* extract microsphere (MEM) is also optimized using the Box-Behnken design. The formulation was prepared at optimized conditions (chitosan concentration (1.66% w/w), volume of glutaraldehyde (4.69 mL), and stirrer rotation per minute, RPM, 854.8), and the percentage yield (*Y*_1_) of the resulted microspheres is ∼95% with an encapsulation efficiency (EE) of (*Y*_2(rutin)_) ∼86%, *Y*_2(quercetin)_) ∼85%, and particle size (*Y*_3_) of ∼40 *µ*m. The MEM prepared at optimized conditions can also be characterized for various parameters to ensure the uniformity of parameters. Also, the drug release studies indicated that the percentage release of rutin and quercetin from MEM was enhanced as compared to *M. alba* extract (ME) alone. Furthermore, *in vivo* analysis of the antiulcer potential of pretreatment with ME and MEM (500 mg/kg p.o.) in rats indicated that mucosal lesions, gastric juice volume, and total acidity were significantly altered as compared to ethanol-treated animals. Histopathology of tissue sections also confirmed the protection of gastric mucosa on pretreatment with MEM at 500 mg/kg p.o. On the basis of these findings, we can conclude that prepared microspheres can be used to develop a sustained release formulation of extract for the management of gastric ulcers. However, additional research is needed to establish the specific mechanisms of *M. alba's* antiulcer efficacy.

## 1. Introduction

Peptic ulcer disease is among the most frequent digestive disorders in the 21^st^ millennium [1]. Peptic ulceration represents isolated, long-term sores that can appear anywhere in the gastrointestinal tract [2]. In the United States, there is a 10% lifetime risk of developing peptic ulcer disease. The complex pathophysiology of this sickness was triggered by a disparity between belligerent drives such as acid and pepsin, on the single reach, and mucous membrane defence elements like blood flow and prostaglandins, on the other [3]. Peptic ulcer disease (PUD) can be caused by a variety of causes, “including stress, alcohol consumption, smoking, *Helicobacter pylori* infection, and the use of nonsteroidal anti-inflammatory drugs (NSAIDs) [4]. Although antibiotics, proton pump inhibitors (omeprazole), prostaglandin analogues, and H2 receptor blockers (cimetidine, ranitidine, and famotidine) reduce the mortality of stomach ulcers, yet more research is needed to find novel medications that are less expensive and have fewer adverse effects [5]. Herbal products are a major part of formulations for preventing and treating digestive problems [6]. One of these is the extract from the leaves of *Morus alba* L. (Family- Moraceae). *M. alba* is also known as white mulberry and is widely used in the preparation of traditional formulations in various countries such as India, China, and Japan [7]. The various proteins, crude fibres, neutral dietary fibres like moran 20 K and isoprene substituted flavanones such as kuwanon G and kuwanon C″ are found in the plant's leaves [8–10]. The different flavonol glycosides such as mulberrofuran G, Albanol B, quercetin 3-(6-malonylglucoside), rutin, isoquercitrin, and astragalin are also reported in the plant [11–13]. Owing to the presence of various constituents, including the different flavonoids, the leaf extract of mulberry has been extensively reported as a potential antioxidant which helps in the management of different diseases [14–18]. Moreover, the plant extract also possesses the antibacterial properties and reduced the IL-1,6 and TNF-*α* production which indicated the anti-inflammatory potential of the herb [19–22]. On the basis of literature, the extract of leaves of *M. alba* L. (ME) is selected for evaluation of protection against ethanol-induced gastric ulcers. Moreover, the formulations such as floating microspheres, which are small-sized granules and float on the gastric fluids, thus release the content slowly for an extended period of time and improve the bioavailability of drugs. Also, the reduction in the daily dose of floating microspheres boosts patient compliance [23–25]. Moreover, the application of response surface methodology (RSM) based on the design of experiments (DoE) could further reduce the number of experiments to get the optimized conditions for the preparation of formulations. Moreover, the technology can be used to optimize the effect of various independent variables on the selected responses. This is the more efficient and cost-effective methodology as compared to the single factor analysis [26–29].

Hence, in the present study, the floating microspheres of the leaf extract of *M. alba* L. are prepared and optimized for three independent variables (factors), namely the concentration of polymer, cross-linking agent, and stirring speed using the Box-Behnken design. Also, the optimized formulation (*M. alba* extract microsphere, MEM) was evaluated for antiulcer potential in experimental Wistar rats.

## 2. Materials and Methods

### 2.1. Plant Material

Fresh mulberry (*Morus alba*, MA) leaves were collected from the herbal garden of the Vaish Institute of Pharmaceutical Education and Research, Rohtak, Haryana, India. The plant samples were taxonomically recognized and validated by a botanist before the start of research work.

### 2.2. Reagents and Chemicals

The various reagents and chemicals such as chitosan, acetic acid, and ethanol were purchased from Sigma-Aldrich Chemical Limited (St. Louis, MO, USA); glutaraldehyde from Acuro Organics Limited (New Delhi, India); Span 80 from Lobachemie (Mumbai, Maharashtra, India); carboxymethyl cellulose from Paras Enterprises (Mumbai, Maharashtra, India); Ketamine from Troikaa Pharmaceuticals Limited (Ahmedabad, Gujarat, India); and xylazine from Alivira Animal Health (Thane, Maharashtra, India).

### 2.3. Preparation of Extract

The fresh leaves of the selected herb were cleaned, washed, and dried at room temperature. The dried plant part was pulverized into a fine powder and sieved (20 mesh), and stored in an airtight glass container. For the preparation of the extract, 20 g of dried leaves powder were extracted for 1 h using ethanol in a Soxhlet apparatus. The extract was concentrated to remove the traces of solvent, and the dried extract was ethanol kept in an airtight container till further use [30].

### 2.4. Preparation of Chitosan Microspheres

Chitosan was dissolved in 10 mL of 5% aqueous acetic acid, and to this solution, the extract solution was added and gently emulsified with light liquid paraffin (100 mL). The solution was mixed for about 5 min using a magnetic stirrer at various stirring rates. In various ratios, glutaraldehyde (GA) was added to this *w*/*o* emulsion as a cross-linking agent, and the mixture was stirred for 2 h. Furthermore, to remove liquid paraffin, unreacted GA, and adhering surfactants, the microspheres were vacuum filtered before being washed with petroleum ether and water. Finally, the prepared solid microspheres were dried at 50°C for 24 h and kept in a desiccator [31, 32]. The percentage yield of MEM was calculated using the following formula:

(1) $$\begin{array}{c} \text{percentage yield}=\frac{\text{practical amount}}{\text{theoretical amount}}\times 100. \end{array}$$

Furthermore, the particle size of the developed microspheres was determined with the help of different micrometers [33]. Moreover, the entrapment efficiency (EE) for rutin and quercetin in MEM was also calculated. For the determination of EE, the MEM (100 mg) was crushed in a pestle and mortar and dissolved in ethanol. The solution is filtered, and the filtrate was diluted and analyzed for concentration of rutin and quercetin by the previously developed HPLC (RP-HPLC, Shimadzu, Japan) method. The % EE was calculated using the following formulae:

(2) $$\begin{array}{c} \%\;EE=\frac{\text{amount of rutin or quercetin in microspheres}}{\text{amount of rutin or quercetin in extract added for MEM}}\times 100. \end{array}$$

Consequently, the loading of rutin or quercetin with respect to the total weight of microspheres, MEM, was also calculated as follows:

(3) $$\begin{array}{c} \text{drug }l\text{oading}\left(\%\right)=\frac{\text{rutin or }q\text{uercetin in }m\text{icrospheres}}{\text{total }w\text{eight of }m\text{icrospheres}}\times 100. \end{array}$$

### 2.5. Optimization of Formulation (Microsphere) Process

From the results of trial batches, it was observed that the concentration of chitosan, glutaraldehyde, and stirrer speed (RPM) were the most critical factors for the development of extract-loaded microspheres. Thus, the process of the formulation of MEM was optimized using the Box-Behnken design (BBD) coupled with response surface methodology (RSM). The different process variables such as concentration of selected polymer, chitosan (*A*), the volume of cross-linking agent, glutaraldehyde (*B*) and stirring speed in rotation per minute, RPM, (*C*) were optimized for the responses such as percentage yield (*Y*_1_), entrapment efficiency for rutin (*Y*_2a_) and quercetin (*Y*_2b_) and particle size of microsphere (*Y*_3_). The actual values for the independent variables are given in Table 1. A total of seventeen experiments, as suggested by design, were carried out, which represented the six axial points, eight factorial points and three central points. Furthermore, a polynomial equation (equation (4)) was used to study the linear, interactive, and quadratic effects of process variables on responses [34].

(4) $$\begin{array}{c} \left(Y\right)=\beta_{0}+\sum_{i=1}^{n}\beta_{i}x_{i}+\sum_{i=1}^{n}\beta_{ii}x_{i}^{2}+\sum_{i\neq j}^{n}\beta_{ii}x_{i}x_{j}+\varepsilon . \end{array}$$

Moreover, the significance of the obtained data was determined using analysis of variance (ANOVA). Also, to test the suitability of the developed model to construct the response surfaces, the design expert software (7.0.3, Stat-Ease, Inc, Minneapolis, USA, trial version) was employed. Consequently, a desirability function that makes use of the numerical optimization technique simultaneously optimized each response and was used to select the optimized conditions for the development of MEM [35]. Finally, the MEM was also developed at the optimized conditions and percentage yield, EE for rutin and quercetin and particle size were determined to validate the predicted responses.

### 2.6. Evaluation of Developed Optimized Formulation

The MEM formulated at optimized conditions were further evaluated for scanning electron microscopy (SEM), floating/buoyancy capacity, *in vitro* release studies, and the ability to protect against gastric ulcers in experimental animals. Before that, the compatibility study of MEM with *M. alba* extract (ME) and physical mixtures of extract with different excipients were also performed using a Fourier-transform infra red (FTIR) spectrophotometer (Shimadzu, Japan), differential scanning colorimetry (DSC) (DSC-60 plus, Shimadzu, Japan) and X-ray diffractometer (XRD) analysis. For FTIR analysis, the samples were mixed with potassium bromide (KBr) and pellets were formed. These pellets were further analyzed in the wavelength range of 4000-400 cm^−1^ [36]. Furthermore, for DSC analysis, the samples were carefully weighed and heated in closed aluminium crimped cells at a rate of 10°C·min^−1^ between 30° and 300°C under a nitrogen flow of 40 mL/min [37]. Moreover, the XRD patterns were achieved using an X-ray diffractometer (ARL Equinox 100, Thermofisher Scientific, India). The diffraction angle scan range used was 0–500 degrees. The XRD pattern was measured using a voltage of 40 kV and a current of 30 mA [37].

### 2.7. SEM Analysis of MEM

Optimized microspheres were dried overnight and examined under a scanning electron microscope (JEOL JSM-6480LV, Japan). In this process, a focused electron beam was employed to scan the material in parallel lines. Microspheres were then sputter coated with a conducting metal such as platinum or zirconium and placed on a sample holder for SEM analysis. A tightly focused electron beam was then used to scan the material. The surface parameters of the sample were determined using secondary electrons emitted from the sample surface [38].

### 2.8. Floating/Buoyancy Capacity of MEM

100 mg of developed MEM were placed in 250 mL of 0.1 N HCl. The mixture was swirled at 100 rpm in a magnetic stirrer with magnetic beads. After 24 h, the layer of buoyant floating microspheres was removed and filtered. For both fractions of microspheres, the weight ratio of floating particles to the addition of floating and settled MEM was utilised to determine the buoyancy of the developed MEM [39–41].

(5) $$\begin{array}{c} \%b\text{uoyancy}=\frac{\text{weight of }f\text{loating MEM}}{\left(\text{weight of }f\text{loating MEM}+w\text{eight of }s\text{ettled MEM}\right)}\times 100. \end{array}$$

### 2.9. In Vitro Drug Release Studies and Kinetics

The *in vitro* drug release profile of MEM was carried out using a USP dissolving equipment I basket type (Model DS-8000 LabIndia). The capsules were filled with 500 mg of MEM and placed in the dissolving basket containing 0.1 N HCl (500 mL) rotating at a speed of 100 rpm and operating at a temperature of 37.5°C. About 2 mL of aliquots were withdrawn from the dissolving media at different times and replaced with a new buffer solution at regular intervals. The samples were filtered using a 0.45 *µ*m membrane filter, and the amounts of rutin and quercetin were determined using a predeveloped HPLC method. After determining the amount of drug in the filtrate, the percent drug release was calculated [42]. Furthermore, to characterize the release profile, an appropriate model mathematical function was selected and evaluated using the derived model parameters. Different rate kinetic models were used to depict the results of *in vitro* release studies [43].

### 2.10. Evaluation of Antiulcer Potential

The protective effect of prepared MEM against gastric ulcer was evaluated in experimental rats. The research protocol for experiments on the animals was approved by the Institutional Animal Ethical Committee of Maharshi Dayanand University vide reference number 1767/GO/ReS/14/CPCSEA, 76–85, dated 26/02/2021. Briefly, the experimental protocol is described as follows.

#### 2.10.1. Acute Oral Toxicity Study

The standard OECD guidelines were followed to determine the acute oral toxicity of plant extracts. For this, two groups of animals (*n* = 6) were formed. One received the carboxyl methyl cellulose (1%) in normal saline and the other was given the plant extract in different doses (50, 500, and 5000 mg/kg) by oral route. The rats were observed regularly for any significant behavioral changes, general motor signs, and mortality till 72 h [44].

#### 2.10.2. Experimental Protocol

Experimental animals (Wistar rats) with an average body weight of 200–250 g were used for the present study. Animals were housed in conventional cages with free access to water and feed during the experiments. The rats were divided into five groups (*n* = 6) and the experimental protocol lasted up to seven days. The first group of animals (control) received normal saline for seven days, while the second and third groups received alcohol (5 ml/kg, negative control) and omeprazole (20 mg/kg, positive control), respectively. The fourth and fifth groups were termed as test groups and were fed with MA extract and MEM solution, respectively, at a dose of 500 mg/kg [44, 45]. On the last day, rats of all test groups, including a positive control, were given absolute ethanol (5 ml/kg b.w.) to produce gastric ulcers [46]. After 2 h, the animals were euthanized by cervical dislocation under anesthesia of ketamine (80–100 mg/kg) and xylazine (10–12.5 mg/kg) in normal saline through an intraperitoneal route, and their stomachs were collected. The specimen of the stomach wall was stained with haematoxylin and eosin dye to prepare the sections for microscopical examination [47]. Furthermore, the gastric ulcer index (GUI), volume and pH of gastric fluid, and total acidity were also calculated with the following methods.

#### 2.10.3. The Gastric Ulcer Index (GUI)

To evaluate gross lesions, the stomach clumps were opened along the anterior surface, rinsed and washed with cold normal saline, blotted dry between filter paper sheets, and pinned flat on cardboard to determine the GUI using the Guth et al. technique [48]. Each gastrointestinal cavity was thoroughly examined, and ulceration severity was assessed and scored as “0,” if there were no lesions (normal stomach); 0.5, hyperaemia (red colour); 1, haemorrhagic patches; 2, 1–5 tiny ulcers; 3, many small ulcers; 4, numerous small and big ulcers; and 6, stomach full of perforated ulcers [49, 50]. Also, the protective index (PI) was computed using the following equation:

(6) $$\begin{array}{c} PI=\frac{UI\left(\text{ulcerated}\right)-UI\left(\text{pretreated}\right)}{UI\left(\text{ulcerated}\right)}\times 100. \end{array}$$

#### 2.10.4. Gastric Volume and pH Determination

For the determination of gastric volume, the stomach content was dumped into tubes and centrifuged for 10 min at 1000 rpm. Furthermore, the pH of the content was also determined after diluting the 1 mL of gastric juice with 1 mL of distilled water [47].

#### 2.10.5. Determination of Total Acidity

An aliquot of (1 mL) gastric fluid was blended with 1 mL of distilled water and two drops of phenolphthalein indicator were added. The prepared solution was titrated with NaOH (0.01 N) until a persistent pink colour was observed [47]. The acidity was expressed as mEq/L by the following formula:

(7) $$\begin{array}{c} \text{total }a\text{cidity}=v\text{olume of NaOH}\times N\times 100, \end{array}$$

*N* = normality.

#### 2.10.6. Statistical Analysis

The statistical significance of the observed data was determined using the GraphPad Prism 9 software (CA, United States). The data were analyzed using the one-way analysis of variance (ANOVA) followed by Dunnett's test. All the data were represented as mean ± standard deviation (S.D.) and results with a probability (*p* value) less than 0.05 was considered to be significant.

## 3. Results and Discussion

*Morus alba* L. is commonly known as the mulberry plant, and the leaves of this herb are generally used in Asian countries as functional foods for the prevention and treatment of various diseases [51]. The various secondary metabolites such as phenolic acids (caffeic acid, ferulic acid, chlorogenic acid), flavonols like 3-O- rutinoside and quercetin 3-*β*-D glucoside, etc. are reported in the leaves of the selected herb and are proved to be responsible for the antioxidant potential of the herb [52, 53]. Various reactive oxygen species (ROS) produce oxidative stress and initiate the pathophysiology of different diseases, including gastric ulcers [54]. Thus, in the present work, an extraction of the leaves of *M. alba* L. was selected for the evaluation of the antiulcer activity. Moreover, the successful treatment of gastric ulcers also requires enhanced gastric residence time so that the drug can be infringed into the submucosa region of the stomach for better action. But conventional dosage forms like tablets and capsules pass through the stomach very quickly due to their high weight and, thus, require high dosage and possess poor patient compliance [55]. Hence, for the present research, we aimed at the preparation of a floating microsphere of MA leaves extract, which being lighter in weight could float in the gastric fluid and could provide sustained release of the plant actives to the targeted mucosa for a longer time. Consequently, various species of the genus *Morus* are available in India . Hence, to confirm the identity of the collected sample, first it was authenticated by a botanist on the basis of morphological characters. Also, a specimen sample was kept in the department for future reference. Furthermore, the leaves were extracted with the selected solvent, and the extract yield was found to be 29.8% (w/w). Consequently, to confirm the presence of polyphenolic constituents (rutin and quercetin) of therapeutic significance, the HPLC analysis of the extract was carried out by a previously developed RP-HPLC method in our laboratory. The concentration of rutin and quercetin was found to be 0.43 ± 0.12% and 0.63 ± 0.21%, respectively. In comparison with past results, it was found that the rutin concentration was almost half, whereas the quercetin was about three times the previously reported yield [56]. Different geographical sources and environmental conditions of the raw material severely affect the concentration of secondary metabolites and could also be the reason for variation in the present case [57]. Furthermore, the extract was kept in an airtight container until the preparation of microspheres.

### 3.1. Preparation of Extract Microspheres

Different factors, such as concentration of polymer, cross-linking agent, surfactant, and process variables like stirrer speed, etc., significantly influence the development of microspheres. For the present research, chitosan was selected as the polymer as it is a naturally derived polysaccharide and has been enormously used in the past for the development of sustained release delivery systems. Moreover, it can also act under mild pH conditions and is safe as compared to synthetic ones [36, 58]. Consequently, some preliminary batches were prepared to select the effective variables and their range for the optimization and development of microspheres. The various batches with different concentrations of chitosan (0.5–2.5%), surfactant, Span 80 (0.5–1.5%), glutaraldehyde (1–7 mL), and RPM (700–1600) were prepared and evaluated on particle size and drug loading efficiency (Tables S1–S4). It was found that the average particle size (∼59 *µ*m) is higher and the loading capacity (∼0.18%) is less for varying concentrations of surfactant as compared to microspheres prepared by altering other variables. Results of these trials also indicated the significant effects of polymer, cross-linking agent, and stirrer speed (RPM) on the preparation of *M. alba* extract microspheres (MEM). Thus, these parameters were selected for further optimization studies.

### 3.2. Model Fitting for MEM

Application of Box-Behnken design suggested seventeen experiments at different conditions of selected variables. The responses (%yield, entrapment efficiency for rutin/quercetin, and particle size) of various experiments are presented in Table 2. Results indicated that the percentage yield (*Y*_1_) of MEM in ten batches was more than 85% and it ranged between 85.56 to 95.46 percent for all batches. Similarly, the % EE for rutin and quercetin (*Y*_2a_ and *Y*_2b_) was found to be between 75.95–87.71% and 73.90–84.88%, respectively, whereas the particle size (*Y*_3_) was determined in the range of 38.27–45.09 *µ*m.

Consequently, the Design-expert software analyzed the results and predicted the polynomial quadratic equation to confirm the effect of independent variables on the various responses. Also, the magnitude of the effect of different factors on dependent variables was predicted by developing a polynomial equation for each response (equations (8)–(11)).

(8) $$\begin{array}{c} Y_{1}=90.32+1.07A+0.96B-6.31C-0.070AB+2.08AC+0.018BC-3.13A^{2}-0.94B^{2}+1.54C^{2}, \end{array}$$

(9) $$\begin{array}{c} Y_{2a}=80.36+1.90A+1.97B-4.70C-0.61AB+0.11AC+0.23BC-3.18A^{2}-0.95B^{2}+5.70C^{2}, \end{array}$$

(10) $$\begin{array}{c} Y_{2b}=77.76+1.66A+1.48B-4.47C+0.37AB-0.52AC-0.26BC-2.78A^{2}-1.11B^{2}+5.66C^{2}, \end{array}$$

(11) $$\begin{array}{c} Y_{3}=39.95+2.46A-2.62B-0.66C-0.66AB-0.55AC+0.48BC+3.79A^{2}+2.08B^{2}+1.15C^{2}. \end{array}$$

Furthermore, the analysis of variance (ANOVA) was applied to the obtained data to get the significance of the results (Table 3). It was found that the developed model is significant (*p* < 0.001) for all the responses and can predict the experimental conditions successfully. Moreover, the lack of fit value (*p* > 0.05) also justified the suitability of the developed model. Additionally, the software also predicted that the value of the coefficient of determination (*R*^2^) for all the factors is close to one (*R*^2^ > 0.9), which confirms that the independent and dependent variables are sufficiently corelated [59]. Moreover, the software also predicted the adequate precision (*Y*_1_-62.06, *Y*_2a_-29.14, *Y*_2b_-48.51, *Y*_3_-31.36) for different responses, which refers to the signal-to-noise ratio and is a measure of a possible error in the anticipated response range. A ratio of more than 4 for all the responses indicated the presence of appropriate and sufficient signal for each response, and a developed model can be used to navigate the design space [60].

**Figure 1:**
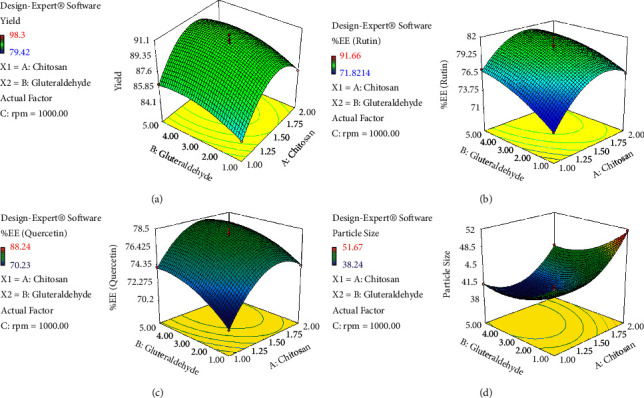
A3D graph indicating the effects of chitosan (A) and glutaraldehyde (B) on various responses: (a) %yield, (b) %EE (rutin), (c) %EE (quercetin), and (d) particle size.

**Figure 2:**
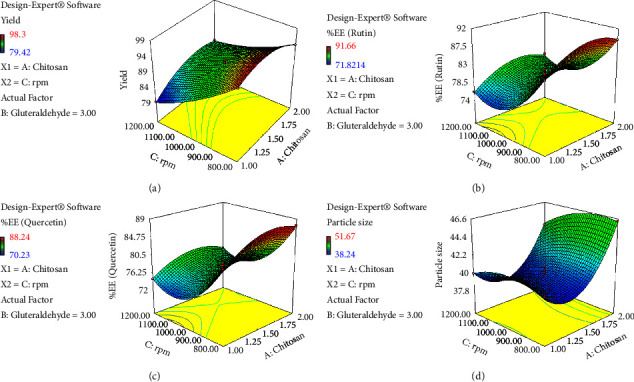
A 3D graph indicating the effects of chitosan (A) and RPM (C) on various responses: (a) %yield, (b) %EE (rutin), (c) %EE (quercetin), and (d) particle size.

**Figure 3:**
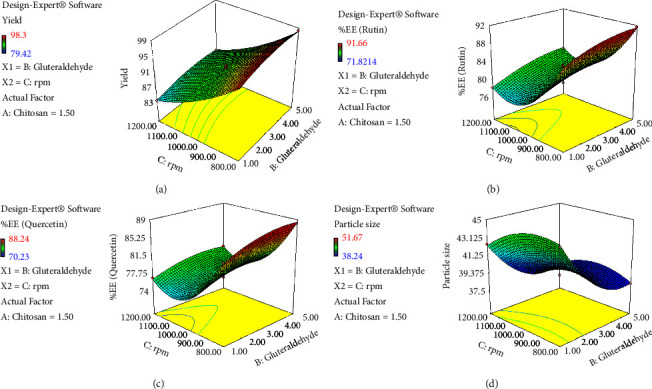
A 3D graph indicating the effects of glutaraldehyde (B) and RPM (C) on various responses: (a) %yield, (b) %EE (rutin), (c) %EE (quercetin), and (d) particle size.

Furthermore, the data analysis also indicated that the effect of linear and square terms for all the variables is significant (*p* < 0.005) except B^2^ for *Y*_2a_ and *Y*_2b_, whereas the interactive terms for all factors are not significant except AC (*p* < 0.001) for *Y*_1_ (Tables S7–S10). Consequently, the design expert software was also predicted to draw the 3-D graph indicating the effect of different variables on selected responses (Figures 1–3). These graphs are quite important for understanding how a change in the level of one component affects the effect of another. At least one independent variable must always be fixed because these graphs can only represent two or more independent variables versus the reaction at a time [61].

### 3.3. Optimization and Validation of the Development Model

The experimental conditions for the development of MEM were further optimized using a numerical optimization approach. This approach suggested different solutions and optimized conditions for the preparation of MEM. The conditions were selected on the basis of criteria like minimum particle size and highest EE and percentage yield. Design-expert software predicted the chitosan concentration of 1.66% (w/w), 4.69 mL of glutaraldehyde, and a stirrer speed of nearly 854 RPM for the development of MEM. Moreover, the experiments were also carried out at the predicted conditions to validate the optimized conditions. Consequently, it has been observed that response values for dependent variables are within the 95 percent confidence limit (Table S11) and thus, validates the developed model for the preparation of MEM.

### 3.4. Evaluation of Optimized Formulation

**Figure 4:**
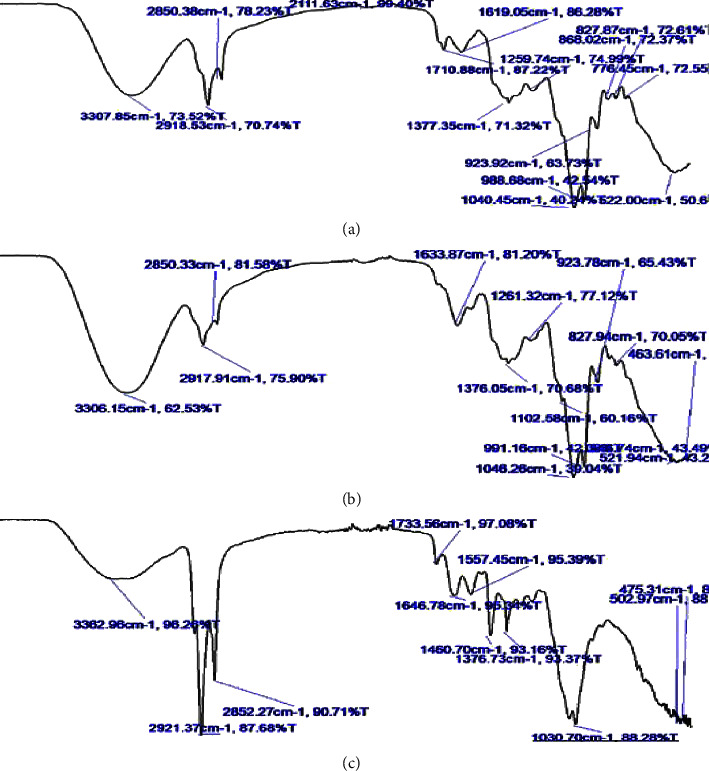
The FTIR spectra of (a) mulberry extract, (b) physical mixture (extract, chitosan, GA, and span80), and (c) optimized formulation.

The prepared microspheres of the extract at optimized conditions were evaluated on various parameters. The particle size of MEM was found to be 40.1 ± 0.07 *µ*m and the % EE for rutin and quercetin was analyzed to be 86.93 ± 0.55 and 85.09 ± 0.15%, respectively. Furthermore, the loading capacity of rutin and quercetin was evaluated to be 0.237 ± 0.09 and 0.249 ± 0.021%, respectively. Furthermore, the optimized formulation was processed for compatibility studies with respect to extracts and excipients by FTIR, DSC, and XRD analysis. The FTIR spectra of mulberry leaf extract showed O-H vibration at 3307.85, primary and secondary amine at 1619.05, C-N vibration stretch at 1377.35, C-O stretch at 1040.45, C-O- and C-OH stretching at 988.68 and C-C skeleton vibration at 522.0 cm^−1^ (Figure 4(a)).The spectra of the physical mixture i.e., extract, chitosan, GA and span80, represented the absorption band at 3306.15 cm^−1^ (-OH vibration stretching of alcoholic and phenolic compounds), 1633.87 cm^−1^ (primary and secondary amines), 1376.05 cm^−1^(C-N vibration stretch), C-O stretch at 1046.26, C-O- and C-OH stretching at 991.16 and C-C skeleton vibration at 521.94 cm^−1^ (Figure 4(b)). Retention of the characteristic peaks of the drug i.e., O-H vibration (3362.96 cm^−1^), primary and secondary amine (1646.77 cm^−1^), and C-N vibration stretch at (1376.7 cm^−1^) in the FTIR spectra of optimized formulation revealed the compatibility of the drug and chosen excipients (Figure 4(c)). Furthermore, the absence of other peaks and no significant movement of existing peaks indicated that the extract was successfully incorporated into the microspheres [62].

**Figure 5:**
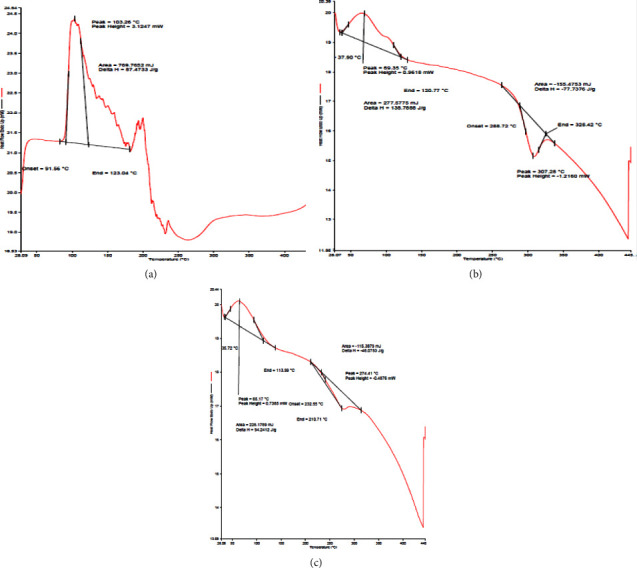
DSC thermogram of (a) mulberry extract, (b) physical mixture (extract, chitosan, GA, and Span80), and (c) optimized formulation.

In the DSC curve of mulberry extract, a broad endothermic peak (*T*_onset_ = 91.56°C; *T*_peak_ = 103.26°C) was observed (Figure 5(a)). However, in the case of the physical mixture, the DSC thermogram indicated two broad endothermic peaks, one being (*T*_onset_ = 37.90°C; *T*_peak_ = 69.35°C) and another one (*T*_onset_ = 288.72°C; *T*_peak_ = 307.25°C) (Figure 5(b)). As both the drug and polymer peaks are still visible, they demonstrate that neither of the two components changes its thermal behaviour in relation to the raw ingredients. However, the thermal curves of optimized formulation showed a shift in endothermic peaks (Figure 5(c)), one being at (*T*_onset_ = 35.72°C; *T*_peak_ = 65.17°C) and another one at (*T*_onset_ = 232.55.72°C; *T*_peak_ = 274.41°C). The absence of the peaks typical of *M. alba* extract in the MEM demonstrated a significant alteration in the extract's thermal behaviour. According to the DSC findings, we can conclude that the extract was included in the chitosan polymeric network and was totally amorphized [63].

**Figure 6:**
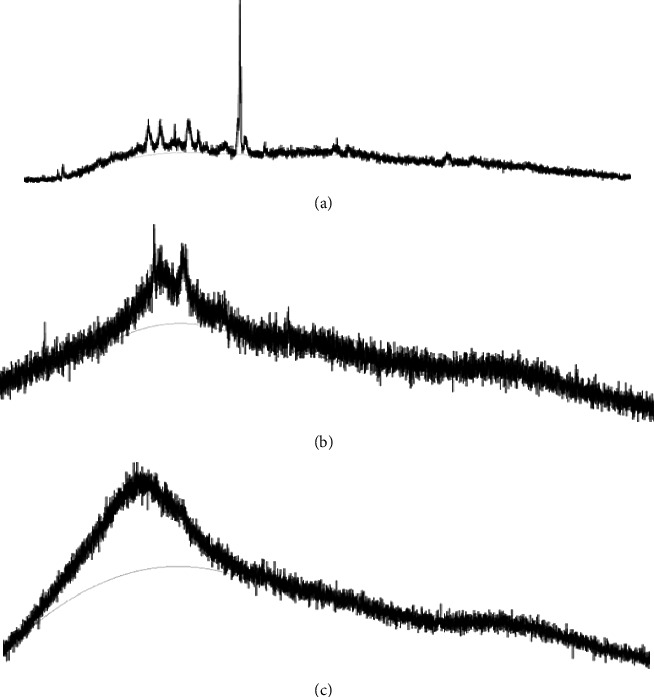
X-ray diffractogram of (a) mulberry extract, (b) physical mixture (extract, chitosan, GA, and Span80), and (c) optimized formulation.

The X-ray diffraction studies of mulberry extract, physical mixture, and optimized formulation are shown in Figures 6(a)–6(c). In the case of mulberry extract, the characteristic peaks of quercetin exhibited at a diffraction angle of 2*θ*, 10.731°, 12.338°, 15.872°, 24.414°, 26.501°, and 27.407° as well as rutin powder sharp peaks at a diffraction angle of 2*θ*, 11.2°, 14.8°, 16.2°, and 26° were present and it can be inferred to traits of a high crystalline structure (Figure 6(a)). The peaks of chitosan were seen at 2*θ* values of 11.703° and 20.297°, which were in good agreement with the values from the literature [58]. The broadening of the peak in Figure 6(b) also confirmed the amorphous nature of the polymer. Consequently, the XRD pattern of the developed MEM did not indicate these peaks, and hence, confirmed that ME was either molecularly dispersed in the polymer or disseminated in an amorphous state (Figure 6(c)) [64].

Furthermore, the SEM analysis of the optimized formulation was carried out along with the floating ability and in vitro release studies.

### 3.5. SEM Analysis

**Figure 7:**
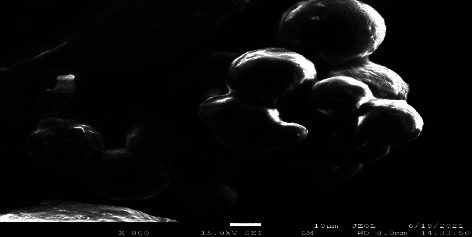
A scanning electron microscopy (SEM) of an optimized formulation (MEM).

The prepared microspheres were analyzed for surface morphology by SEM, and the results are depicted in Figure 7. It indicated the typical images of the microspheres under the standard preparative conditions and their enlarged surface structures. It can be seen that the microspheres were spherical in shape and possessed regular geometry with a smooth surface [65].

### 3.6. Floating Ability of MEM

The floating ability of prepared microspheres, MEM, was analyzed and the results indicated the buoyancy capacity of the optimized formulation is about 86.19 ± 2.9%. The purpose of the floating test was to see if the prepared microspheres could float in gastric fluid or not. The fraction of microspheres that settled down as a function of time was measured after the microspheres were distributed over the surface of the buffer medium. In the present study, the chitosan-based optimized formulation demonstrated good floating ability for about 24 h. The hollow nature of the microspheres is likely to be responsible for their good buoyancy behaviour [66].

### 3.7. In Vitro Release of Rutin and Quercetin

**Figure 8:**
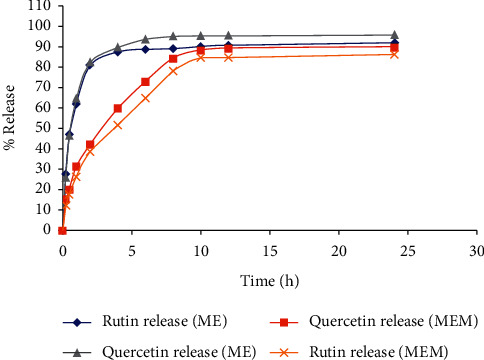
(%) release of rutin and quercetin from *M. alba* extract (ME) and optimized formulation (MEM).

The *in vitro* release of mulberry leaves extract and prepared MEM for rutin and quercetin was analyzed. The results indicated that about 80% of the plant actives were released within two hours of the extract, whereas a similar concentration of marker compounds was analyzed in the samples after eight hours (Figure 8). These findings indicate that prepared floating microspheres can effectively enhance the gastric residence time and would be suitable for the development of a sustained drug delivery system of ME [67, 68].

Further, the release kinetics study (Figure S5) indicated that the optimized formulation follows the Korsmeyer-Peppas model (*R*^2^ > 0.95). Moreover, the kinetics of the formulation showed no burst effect as compared to the extract alone, which further confirmed the sustained release of plant actives from the prepared chitosan microspheres.

### 3.8. Evaluation of Antiulcer Potential

In spite of significant breakthroughs in the treatment of stomach ulcers, the condition still has a high prevalence [69]. Past investigations on the plants have revealed that extracts with phenolic compounds play a significant role in stomach ulcer prevention [70]. But the formulations of the extracts, especially the tablets, were not found to be very effective in ulcer management. This could be due to the shorter gastric residence period of such formulations and the plant actives are not absorbed effectively through the gastric mucosa to produce the healing effect [71]. The development of floating microspheres of pharmacologically effective extracts could be one such approach that could prolong the retention of extracts in gastric fluid for a sufficient period to get absorbed through the mucosa and thus could help in the management of ulcers [72]. Also, the flavonoids present in the *M. alba* leaves were also reported to possess the antioxidant as well as the anti-inflammatory potential [73]. Moreover, oxidative stress and proinflammatory cytokines were the significant factors behind the occurrence of gastric ulcers [54, 74]. Thus, in the present research, the developed floating microspheres (MEM) of the selected herb were evaluated for their antiulcer potential in the experimental animals. Firstly, the acute toxicity studies were performed as per standard guidelines and no deaths or changes in skin and hair were seen in rats at given doses of plant extract (50, 500, and 5000 mg/kg body weight). This finding is also supported by a prior *In-Vivo* investigation in which the ME was not found harmful to male and female rats [75]. Furthermore, a subchronic toxicity research in the past also indicated no significant impacts on haematological parameters, as well as no substantial histological irregularities after 60 days of orally given ME [76, 77].

**Figure 9:**
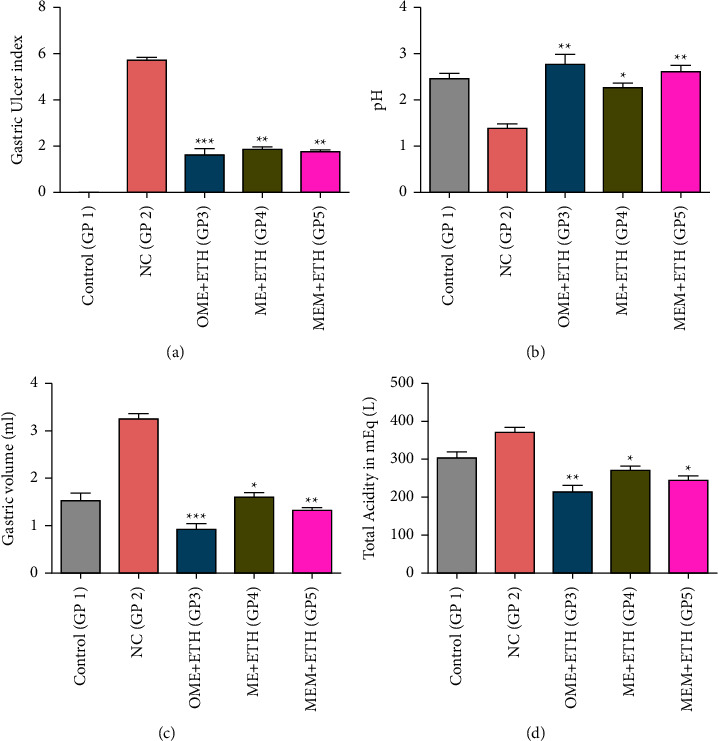
Bar diagram indicating the effect of various parameters: (a) gastric ulcer index (GUI), (b) pH, (c) gastric volume (in mL), and (d) total acidity (mEq/L) in different groups (^*∗*^indicates significance as compare to NC, ^*∗*^*p* < 0.05, ^*∗∗*^*p* < 0.01, ^*∗∗∗*^*p* < 0.005).

The formation of stomach ulcer lesions in rats was induced by pretreatment with ethanol at 5 mL/kg/day. A gastric ulcer caused by ethanol is a typical animal model used to test the antiulcer potential of extracts [78]. With the disruption of the vascular endothelial cells and facilitation of vascular permeability, alcohol boosts acid secretion and lowers blood flow, resulting in microvascular damage. Ethanol also causes cellular antioxidant mechanisms to become unbalanced and induces the generation of superoxide anion and hydroperoxyl-free radicals, resulting in enhanced oxidative stress in the tissues [6, 79]. A study of the isolated stomach of the ethanol-treated animals (negative control, GP2) revealed that the ulcer index, gastric volume, and total acidity were significantly enhanced, whereas the pH was significantly reduced (*p* < 0.01). On treatment with Omeprazole (Positive control, GP3), and ME (GP4), and MEM (GP5), the selected parameters were significantly altered (*p* < 0.05) (Table S12, Figure 9).

**Figure 10:**
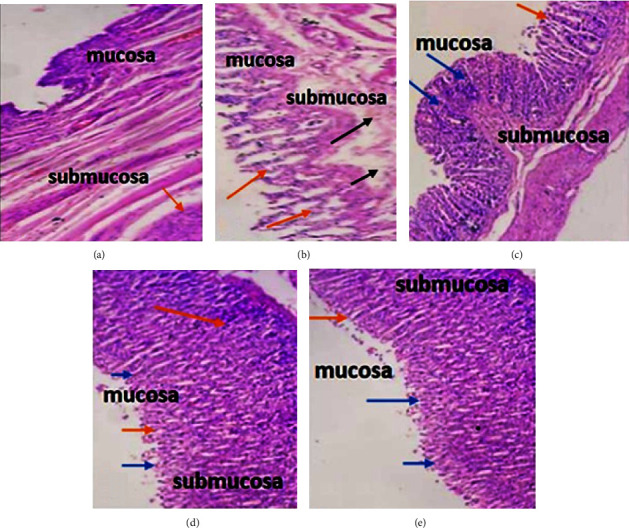
Therapeutic impact of *M. alba* on the lining of the stomach in rats with stomach mucosal damage caused by ethanol. By boosting the stomach mucosa's defensive function, *M. alba* assisted in the recovery of damaged histology. (a) Normal control rats, (b) ethanol-treated rats with profound necrosis and extensive mucosal injury (orange arrow) (black arrow), (c) omeprazole (20 mg/kg + ethanol), (d) ME (500 mg/kg)+ETH, and (e) MEM (equivalent to 500 mg/kg)+ETH showed a potent gastroprotective implication, as evidenced by reduced mucosal aberration, gastric epithelial necrosis, and inflammatory cell infiltration, as well as restoration of the mucosal barrier (blue arrow).

The HPLC analysis of the plant extract revealed the presence of phenolic compounds such as rutin and quercetin in the extract, and these compounds could be responsible for the protective extract of ME and MEM. In the past also such compounds were reported to reduce gastric ulcers by enhancing the prostaglandin content from gastric mucosa, inhibiting the *Helicobacter pylori*, and by scavenging free radicals [80–82]. Furthermore, it was found that in MEM-treated animals, the change in the ulcer index was lower than in ME-treated rats. This could be due to better absorption of extract through microsphere formulation. The chitosan in the formulation was reported to form hydrogen bonds with the sialic acid of mucin and cause the enhanced permeability of drug particles through gastric mucosa [83, 84]. Micrographs of stomach tissue of MEM-treated animals also justified the better absorption of extracts, as the architecture of submucosa cells was significantly improved as compared to ME-treated rats (Figures 10(c)–10(e)). Furthermore, the histopathology of stomach sections from the animals of different groups indicated that ethanol causes gastric lesions, acute degeneration, necrosis, and haemorrhages, as well as considerable worsening of the gastric mucosa (Figures 10(a) and 10(b)). Also, the stomach wall demonstrated significant inflammatory cell infiltration and submucosal swelling. Besides oxidative stress, ethanol also induces the expression of various proinflammatory mediators such as TNF-*α*, IL-1, and IL-6, which are responsible for the damage of the gastric mucosa and submucosa [36]. Treatment with omeprazole, ME, and MEM significantly reduces gastric lesions and alters the gastric mucosa anatomy as compared to the negative control group (Figures 10(c)–10(e)). Further literature revealed that *M. alba* extract, along with the scavenging of free radicals, is also reported to reduce the serum levels of TNF-*α*, IL-1*β,* and IL-4 in experimental animals, which justifies the protection of gastric mucosa from the deteriorating effects of ethanol [85, 86]. Moreover, the protection of gastric submucosa with MEM hints that the enhanced residence time of the floating microsphere in the gastric fluid could be responsible for the better activity of the prepared formulation.

## 4. Conclusion

The mulberry plant, *M. alba* (Family- Moraceae), is commonly used in Asian countries for its various health as well as nutritional benefits. Plant extract is found to be a rich source of phenolic compounds and thus possesses significant pharmacological action in the management of oxidative stress and inflammation. Due to an unhealthy lifestyle, the prevalence of gastric diseases, mainly ulcers, has significantly enhanced and herbal products are commonly used in the treatment of such problems. Moreover, traditional formulations such as tablets rapidly leave the stomach and thus are not very successful in the management of gastric ulcers. Hence, in the present research, we aimed at the development, optimization, and evaluation of the floating microsphere of *M. alba* extract. The present findings indicate the successful preparation of floating microspheres of extract at optimized conditions, and these microspheres also protect the gastric mucosa against ethanol-induced ulcers. Scavenging of free radicals and reduction in different inflammation markers could be the possible reasons behind the antiulcer potential of the extract and its microspheres. Moreover, the *In-Vitro* release studies revealed the prolonged release of phenolic compounds such as rutin and quercetin till 24 h. In a nutshell, we can conclude that prepared microspheres can be used to develop a sustained release formulation of extract for the management of gastric ulcers. However, additional research is needed to establish the specific mechanisms of *M. alba's* antiulcer efficacy.

## Figures and Tables

**Table 1:** Actual values of various independent variables at different levels.

Independent variables	Unit	Actual values	
		−1 level	+1 level
Chitosan concentration (A)	%	1	2
Volume of glutaraldehyde (B)	mL	1	5
Stirrer speed, RPM (C)	RPM	800	1200

**Table 2:** Various runs at different experimental conditions are suggested by the Box-Behnken design.

Run	Independent variables			Responses			
	Chitosan (A)	Glutaraldehyde (B)	RPM (C)	*Y *_1_ (% yield)	*Y *_2a_ (%EE for rutin)	*Y *_2b_ (%EE for quercetin)	Particle size (*µ*m)
R1	1	1	1000	84.14	71.82	70.23	45.25
R2	2	1	1000	86.36	76.54	74.34	51.67
R3	1	5	1000	86.28	77.14	74.13	41.27
R4	2	5	1000	88.22	79.43	76.76	45.07
R5	1	3	800	95.85	85.45	83.11	40.48
R6	2	3	800	93.88	89.33	87.43	46.29
R7	1	3	1200	79.42	76.23	74.87	39.98
R8	2	3	1200	85.77	80.54	77.11	43.61
R9	1.5	1	800	96.5	88.34	84.98	44.48
R10	1.5	5	800	98.3	91.66	88.24	38.33
R11	1.5	1	1200	83.5	78.11	76.89	42.46
R12	1.5	5	1200	85.37	82.34	79.11	38.24
R13	1.5	3	1000	90.1	80.59	77.89	40.03
R14	1.5	3	1000	90.5	79.29	77.02	39.27
R15	1.5	3	1000	90.2	81.87	78.45	40.86
R16	1.5	3	1000	91.01	80.82	78.1	40.36
R17	1.5	3	1000	89.8	79.23	77.32	39.24

**Table 3:** ANOVA applied for the significance of different responses.

Response	*F* value	Probability > *F* (*p* value)	Adjusted *R*^2^	Predicted *R*^2^
Y_1_				
Model	286.2582	<0.0001^a^	0.9938	0.9865
Lack of fit	0.41	0.7528^b^		

**Y**_**2a**_				
Model	58.46335	<0.0001^a^	0.9700	0.9614
Lack of fit	0.14	0.9307^b^		

**Y**_**2b**_				
Model	170.8595	<0.0001^a^	0.9896	0.9805
Lack of fit	0.31	0.8165^b^		

**Y**_**3**_				
Model	69.36264	<0.0001^a^	0.9747	0.9663
Lack of fit	0.15	0.9231^b^		

## Data Availability

The additional data will be made available to the corresponding authors upon reasonable request.

## Supplementary Materials

Supplementary Materials Table S1: composition of microspheres at different ratio of chitosan (extract-250 mg; Span80-1%; GA-1 mL; Liquid paraffin 100 mL; RPM- 700). Table S2: composition of microspheres at different concentrations of Span 80 (chitosan-1.5%; GA-1 mL; liquid paraffin-100 mL; RPM-700). Table S3: composition of microspheres at different concentration of glutaraldehyde, GL (chitosan-1.5%; Span80-1%; liquid paraffin-100 mL; RPM-700). Table S4: composition of microspheres at different stirrer speeds (RPM) (chitosan-1.5%; Span80-1%; GA-1 mL; liquid paraffin 100 mL). Table S5: retention time (RT) and area of rutin and quercetin as analyzed by HPLC. Table S6: concentration of rutin and quercetin in extract. Table S7: significance of different variables for % yield. Table S8: Significance of different variables for %entrapment efficiency (rutin). Table S9: significance of different variables for %entrapment efficiency (quercetin). Table S10: significance of different variables for particle size. Table S11: predicted and observed value of different response along with standard deviations at various optimized solutions. Table S12: evaluation of ME and MEM on the ethanol-induced ulcer model in different groups. Figure S1: HPLC Chromatogram of rutin standard. Figure S2: HPLC chromatogram of quercetin standard. Figure S3: HPLC chromatogram of rutin and quercetin. Figure S4: chromatogram of rutin and quercetin in mulberry extract. Figure S5: (a) zero order, (b) first order, (c) Higuchi release kinetics, and (d) Korsmeyer and Peppas model of optimized formulation (MEM).
