# Supplementary material for: Evaluation of Ulcer Protective Activity of Morus alba L. Extract-Loaded Chitosan Microspheres in Ethanol-Induced Ulcer in Rat Model
Source: Evid Based Complement Alternat Med. 2022 Sep 30;2022:4907585. doi: 10.1155/2022/4907585 (PMC9546716; doi:10.1155/2022/4907585)
Supplement: Supplementary Materials — Table S1: composition of microspheres at different ratio of chitosan (extract-250 mg; Span80-1%; GA-1 mL; Liquid paraffin 100 mL; RPM- 700). Table S2: composition of microspheres at different concentrations of Span 80 (chitosan-1.5%; GA-1 mL; liquid paraffin-100 mL; RPM-700). Table S3: composition of microspheres at different concentration of glutaraldehyde, GL (chitosan-1.5%; Span80-1%; liquid paraffin-100 mL; RPM-700). Table S4: composition of microspheres at different stirrer speeds (RPM) (chitosan-1.5%; Span80-1%; GA-1 mL; liquid paraffin 100 mL). Table S5: retention time (RT) and area of rutin and quercetin as analyzed by HPLC. Table S6: concentration of rutin and quercetin in extract. Table S7: significance of different variables for % yield. Table S8: Significance of different variables for %entrapment efficiency (rutin). Table S9: significance of different variables for %entrapment efficiency (quercetin). Table S10: significance of different variables for particle size. Table S11: predicted and observed value of different response along with standard deviations at various optimized solutions. Table S12: evaluation of ME and MEM on the ethanol-induced ulcer model in different groups. Figure S1: HPLC Chromatogram of rutin standard. Figure S2: HPLC chromatogram of quercetin standard. Figure S3: HPLC chromatogram of rutin and quercetin. Figure S4: chromatogram of rutin and quercetin in mulberry extract. Figure S5: (a) zero order, (b) first order, (c) Higuchi release kinetics, and (d) Korsmeyer and Peppas model of optimized formulation (MEM). [file 4907585.f1.docx]

**TABLE S1: Composition of microspheres at different ratio of Chitosan (Extract-250mg;Span80-1%; GA-1mL; Liquid paraffin 100mL; RPM- 700)**

| **Sr.no.** | **Formulation code** | **Chitosan**  **(%)** | **Size (µm)** | **%yield** | **%EE** | | **%DL** | |
| --- | --- | --- | --- | --- | --- | --- | --- | --- |
|  |  |  |  |  | **R** | **Q** | **R** | **Q** |
|  | F1 | 0.5 | NF | - | - | - | - | - |
|  | F2 | 1 | 50.6±1.505 | 67.25±0.0015 | 40.74±0.069 | 52.21±0.051 | 0.145±0.0002 | 0.2004±0.0002 |
|  | F3 | 1.5 | 45.2±1.316 | 82.84±0.0035 | 82.66±0.123 | 70.18±0.444 | 0.196±0.0003 | 0.270±0.0017 |
|  | F4 | 2 | 54.4±1.349 | 77.33±0.0015 | 67.89±0.369 | 63.94±1.27 | 0.146±0.0008 | 0.249±0.0049 |
|  | F5 | 2.5 | NF | - | - | - | - | - |

**EE-Entrapment efficiency; DL-Drug loading; R-Rutin; Q-Quercetin**

**TABLE S2: Composition of microspheres at different concentration of Span 80 (Chitosan-1.5%;GA-1ml;Liquid paraffin-100mL;RPM-700)**

| **Formulation code** | **Span80** | **Size (µm)** | **%yield** | **%EE** | | **%DL** | |
| --- | --- | --- | --- | --- | --- | --- | --- |
|  |  |  |  | **R** | **Q** | **R** | **Q** |
| F6 | 0.1 | 74.7±1.48 | 77.39±0..25 | 66.94±0.246 | 61.71±0.090 | 0.169±0.0006 | 0.168±0.0002 |
| F7 | 0.5 | 64.5±1.62 | 82.24±0..0031 | 71.64±0.381 | 68.31±0.127 | 0.171±0.0009 | 0.174±0.0003 |
| F8 | 1 | 56±1.41 | 84.78±0.0023 | 79.08±0.410 | 75.99±0.035 | 0.182±0.0009 | 0.188±0.00009 |
| F9 | 1.5 | 61.9±1.81 | 78.06±0.0055 | 69.54±1.35 | 65.58±0.0457 | 0.172±0.0034 | 0.177±0.00001 |

**EE-Entrapment efficiency; DL-Drug loading; R-Rutin; Q-Quercetin**

**TABLE S3:Composition of microspheres at different concentration of Glutraldehyde,GL (Chitosan-1.5%;Span80-1%;Liquid paraffin-100mL;RPM-700)**

| **Formulation code** | **GL** | **Size (µm)** | **%yield** | **%EE** | | **%DL** | |
| --- | --- | --- | --- | --- | --- | --- | --- |
|  |  |  |  | **R** | **Q** | **R** | **Q** |
| F10 | 1 | 56±1.41 | 84.78±0.0023 | 79.08±0.410 | 75.99±0.035 | 0.182±0.0009 | 0.188±0.00009 |
| F11 | 2 | 42.7±0.948 | 96.83±0.0032 | 77.92±0.280 | 76.32±0.636 | 0.236±0.0009 | 0.250±0.0021 |
| F12 | 5 | 41±1.49 | 89.37±0.0036 | 73.06±0.378 | 71±0.871 | 0.205±0.0011 | 0.214±0.0026 |
| F13 | 7 | 48.7±1.33 | 87.83±0.0021 | 67.62±0.26 | 68.60±0.014 | 0.194±0.0007 | 0.212±0.0009 |

**EE-Entrapment efficiency; DL-Drug loading; R-Rutin; Q-Quercetin**

**TABLE S4: Composition of microspheres at different Stirrer speed (RPM) (Chitosan-1.5%; Span80-1%; GA-1mL; Liquid paraffin 100mL)**

| **Sr.no.** | **Formulation code** | **RPM** | **Size (µm)** | **%yield** | **%EE** | | **%DL** | |
| --- | --- | --- | --- | --- | --- | --- | --- | --- |
|  |  |  |  |  | **R** | **Q** | **R** | **Q** |
|  | F14 | 700 | 42.7±0.948 | 96.83±0.0032 | 77.92±0.280 | 76.32±0.636 | 0.236±0.0009 | 0.250±0.0021 |
|  | F15 | 800 | 52.3±1.88 | 94.79±0.0012 | 76.71±0.729 | 71.62±0.146 | 0.203±0.0021 | 0.129±0.0004 |
|  | F16 | 1000 | 45.4±1.83 | 95.29±0.0006 | 80.93±0.845 | 82.01±0.0195 | 0.230±0.0024 | 0.249±0.0011 |
|  | F17 | 1200 | 41.8±1.31 | 91.79±0.0575 | 72.16±0.024 | 70.23±0.0567 | 0.212±0.0021 | 0.221±0.0002 |
|  | F18 | 1600 | 35.4±1.71 | 90.33±0.0552 | 67.17±0.127 | 64.44±0.0945 | 0.201±0.0004 | 0.192±0.0003 |

**EE-Entrapment efficiency; DL-Drug loading; R-Rutin; Q-Quercetin**

**TABLE S5:** Retention time (R_T_) and area of rutin and quercetin as analysed by HPLC

| **S.No.** | **Compound Name** | **Retention time (R_T_)** | **Area** | **Tailing** |
| --- | --- | --- | --- | --- |
| 1 | Rutin | 3.239 | 1414744 | 0.882 |
| 2 | Quercetin | 5.959 | 1019061 | 1.407 |

**TABLE S6: Concentration of Rutin & Quercetin in extract**

| **Compound** | **Area** | **Concentration (mg/25mg of extract)** | **% assay** |
| --- | --- | --- | --- |
| Rutin | 206749 | 0.108235 | 0.4329 |
| Quercetin | 419923 | 0.15896 | 0.63584 |

**TABLE S7:** Significance of different variables for % Yield

| **Source** | **Sum of**  **Squares** | **df** | **Mean**  **Square** | **F**  **Value** | **p-value**  **Prob > F** |  |
| --- | --- | --- | --- | --- | --- | --- |
| Model | 405.9016 | 9 | 45.10018 | 286.2582 | < 0.0001 | significant |
| A-Chitosan | 9.11645 | 1 | 9.11645 | 57.86359 | 0.0001 |  |
| B-Gluteraldehyde | 7.353613 | 1 | 7.353613 | 46.67457 | 0.0002 |  |
| C-rpm | 318.4026 | 1 | 318.4026 | 2020.953 | < 0.0001 |  |
| AB | 0.0196 | 1 | 0.0196 | 0.124404 | 0.7347 |  |
| AC | 17.3056 | 1 | 17.3056 | 109.8415 | < 0.0001 |  |
| BC | 0.001225 | 1 | 0.001225 | 0.007775 | 0.9322 |  |
| A^2^ | 41.24352 | 1 | 41.24352 | 261.7793 | < 0.0001 |  |
| B^2^ | 3.738253 | 1 | 3.738253 | 23.7273 | 0.0018 |  |
| C^2^ | 9.956527 | 1 | 9.956527 | 63.19569 | < 0.0001 |  |
| Residual | 1.102855 | 7 | 0.157551 |  |  |  |
| Lack of Fit | 0.261175 | 3 | 0.087058 | 0.413736 | 0.7528 | not significant |
| Pure Error | 0.84168 | 4 | 0.21042 |  |  |  |
| Cor Total | 407.0045 | 16 |  |  |  |  |

**TABLE S8**: Significance of different variables for %Entrapment efficiency (Rutin)

| **Source** | **Sum of**  **Squares** | **df** | **Mean**  **Square** | **F**  **Value** | **p-value**  **Prob > F** |  |
| --- | --- | --- | --- | --- | --- | --- |
| Model | 412.6243 | 9 | 45.84714 | 58.46335 | < 0.0001 | significant |
| A-Chitosan | 28.89216 | 1 | 28.89216 | 36.8427 | 0.0005 |  |
| B-Gluteraldehyde | 31.02356 | 1 | 31.02356 | 39.56062 | 0.0004 |  |
| C-rpm | 176.3442 | 1 | 176.3442 | 224.8706 | < 0.0001 |  |
| AB | 1.480116 | 1 | 1.480116 | 1.887414 | 0.2119 |  |
| AC | 0.046225 | 1 | 0.046225 | 0.058945 | 0.8151 |  |
| BC | 0.207025 | 1 | 0.207025 | 0.263994 | 0.6232 |  |
| A^2^ | 42.45811 | 1 | 42.45811 | 54.14172 | 0.0002 |  |
| B^2^ | 3.804001 | 1 | 3.804001 | 4.850785 | 0.0635 |  |
| C^2^ | 136.944 | 1 | 136.944 | 174.6283 | < 0.0001 |  |
| Residual | 5.489422 | 7 | 0.784203 |  |  |  |
| Lack of Fit | 0.523022 | 3 | 0.174341 | 0.140416 | 0.9307 | not significant |
| Pure Error | 4.9664 | 4 | 1.2416 |  |  |  |
| Cor Total | 418.1137 | 16 |  |  |  |  |

**TABLE S9:** Significance of different variables for %Entrapment efficiency (quercetin)

| **Source** | **Sum of**  **Squares** | **df** | **Mean**  **Square** | **F**  **Value** | **p-value**  **Prob > F** |  |
| --- | --- | --- | --- | --- | --- | --- |
| Model | 366.0733 | 9 | 40.67481 | 170.8595 | < 0.0001 | significant |
| A-Chitosan | 22.11125 | 1 | 22.11125 | 92.881 | < 0.0001 |  |
| B-Gluteraldehyde | 17.405 | 1 | 17.405 | 73.11182 | < 0.0001 |  |
| C-rpm | 160.0261 | 1 | 160.0261 | 672.2089 | < 0.0001 |  |
| AB | 0.5476 | 1 | 0.5476 | 2.30026 | 0.1731 |  |
| AC | 1.0816 | 1 | 1.0816 | 4.543392 | 0.0705 |  |
| BC | 0.2704 | 1 | 0.2704 | 1.135848 | 0.3219 |  |
| A^2^ | 32.6109 | 1 | 32.6109 | 136.9861 | < 0.0001 |  |
| B^2^ | 5.169112 | 1 | 5.169112 | 21.71348 | 0.0023 |  |
| C^2^ | 134.7438 | 1 | 134.7438 | 566.0077 | < 0.0001 |  |
| Residual | 1.66642 | 7 | 0.23806 |  |  |  |
| Lack of Fit | 0.3167 | 3 | 0.105567 | 0.312855 | 0.8165 | not significant |
| Pure Error | 1.34972 | 4 | 0.33743 |  |  |  |
| Cor Total | 367.7398 | 16 |  |  |  |  |

**TABLE S10:** Significance of different variables for particle size

|  | **Sum of** |  | **Mean** | **F** | **p-value** |  |
| --- | --- | --- | --- | --- | --- | --- |
| **Source** | **Squares** | **df** | **Square** | **Value** | **Prob > F** |  |
| Model | 195.6402 | 9 | 21.73781 | 69.36264 | < 0.0001 | significant |
| A-Chitosan | 48.31445 | 1 | 48.31445 | 154.1654 | < 0.0001 |  |
| B-Gluteraldehyde | 54.86281 | 1 | 54.86281 | 175.0604 | < 0.0001 |  |
| C-rpm | 3.498013 | 1 | 3.498013 | 11.16172 | 0.0124 |  |
| AB | 1.7161 | 1 | 1.7161 | 5.475862 | 0.0518 |  |
| AC | 1.1881 | 1 | 1.1881 | 3.79108 | 0.0926 |  |
| BC | 0.931225 | 1 | 0.931225 | 2.971423 | 0.1284 |  |
| A^2^ | 60.40863 | 1 | 60.40863 | 192.7564 | < 0.0001 |  |
| B^2^ | 18.13332 | 1 | 18.13332 | 57.86116 | 0.0001 |  |
| C^2^ | 5.566 | 1 | 5.566 | 17.76042 | 0.0040 |  |
| Residual | 2.193755 | 7 | 0.313394 |  |  |  |
| Lack of Fit | 0.224675 | 3 | 0.074892 | 0.152135 | 0.9231 | not significant |
| Pure Error | 1.96908 | 4 | 0.49227 |  |  |  |
| Cor Total | 197.834 | 16 |  |  |  |  |

**TABLE S11: Predicted and observed value of different response along with standard deviations at various optimized solutions**

|  | **Predicted value** | | | | **Observed value** | | | | **Standard deviation** | | | |
| --- | --- | --- | --- | --- | --- | --- | --- | --- | --- | --- | --- | --- |
|  | **% Yield** | **%EE (Rutin)** | **%EE (Quercetin)** | **Particle size** | **% Yield** | **%EE (Rutin)** | **%EE (Quercetin)** | **Particle size** |  |  |  |  |
|  |  |  |  |  |  |  |  |  | **% yield** | **%EE (Rutin)** | **% EE (Quercetin)** | **Particle size** |
| DS1 | 88.56 | 75.95 | 73.90 | 44.19 | 90.19 | 78.53 | 74.93 | 46.23 | 1.15 | 1.82 | 0.72 | 1.43 |
| DS2 | 95.43 | 86.48 | 83.73 | 40.74 | 91.31 | 72.51 | 79.49 | 42.11 | 2.91 | 9.88 | 2.99 | 0.96 |
| DS3 | 92.75 | 84.98 | 82.56 | 45.09 | 87.21 | 69.19 | 81.49 | 43.65 | 3.91 | 11.16 | 0.75 | 1.02 |
| DS4 | 95.39 | 87.71 | 84.88 | 39.90 | 95.07 | 86.93 | 85.09 | 40.01 | 0.22 | 0.55 | 0.15 | 0.07 |
| DS5 | 89.32 | 78.18 | 75.77 | 40.57 | 82.86 | 74.69 | 70.36 | 41.76 | 4.56 | 2.46 | 3.82 | 0.83 |
| DS6 | 85.85 | 80.11 | 77.69 | 38.27 | 84.60 | 78.53 | 76.03 | 40.33 | 0.88 | 1.11 | 1.17 | 1.45 |
| DS7 | 90.74 | 81.82 | 79.31 | 44.00 | 87.54 | 84.15 | 80.28 | 46.43 | 2.61 | 1.64 | 0.68 | 1.71 |
| DS8 | 90.38 | 80.27 | 77.61 | 39.09 | 87.98 | 77.11 | 74.84 | 41.32 | 1.69 | 2.23 | 1.95 | 1.57 |
| DS9 | 93.85 | 83.16 | 80.47 | 40.07 | 87.90 | 81.80 | 77.61 | 41.87 | 4.20 | 0.94 | 2.01 | 1.27 |
| DS10 | 92.44 | 81.16 | 78.61 | 40.46 | 88.71 | 79.71 | 77.46 | 41.11 | 2.63 | 1.02 | 0.81 | 0.45 |

TABLE S12: **Evaluation of ME and MEM on ethanol induced ulcer model in different groups**

| **Group** | **Gastric Ulcer index±SD** | **Protection Index±SD** | **pH** | **Gastric volume (ml)** | **Total Acidity in mEq/L** |
| --- | --- | --- | --- | --- | --- |
| Control (GP 1) | 0.00±0.00 | 99.98±0.00 | 2.45±0.12 | 1.52±0.16 | 303 ± 16.76 |
| NC (GP 2) | 5.689±0.05 | 0.01±0.23 | 1.38±0.09 | 3.25±0.11 | 371 ± 12.42 |
| OME+ETH (GP3) | 1.478±0.04*** | 80.5±0.12*** | 2.77±0.21** | 0.78±0.77*** | 215±18.68** |
| ME+ETH (GP4) | 1.83±0.06** | 65.4±0.12*** | 2.28±0.08* | 1.60±0.09* | 271±10.43* |
| MEM+ETH (GP5) | 1.74±0.09** | 74.12±0.18*** | 2.62±0.13** | 1.32±0.05** | 244±11.60* |

*indicates significance as compare to NC, *p<0.05, **p<0.01,***p<0.005

**FIGURE S1: HPLC** Chromatogram of Rutin standard

**FIGURE S2: HPLC** Chromatogram of Quercetin standard

**FIGURE S3:** HPLC chromatogram of Rutin and Qurecetin

**FIGURE S4:** Chromatogram of Rutin and Quercetin in Mulberry extract


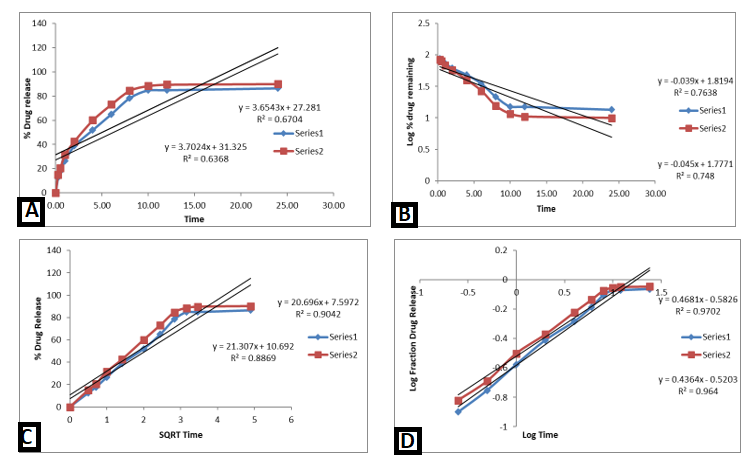


**FIGURE S5: A) Zero order, B) First order, C) Higuchi release kinetics, D) Korsmeyer & Peppas model of optimized formulation (MEM)**
